# Case report: Additional grounds for tighter regulation? A case series of five women with zolpidem dependence from a Brazilian women-specific substance use disorder outpatient service

**DOI:** 10.3389/fpsyt.2024.1456148

**Published:** 2024-12-18

**Authors:** Gabriel Leal, Igor Studart, Caio Petrus Monteiro Figueiredo, Talita di Santi, Paulo Suen, Silvia Brasiliano, Patricia B. Hochgraf, Pedro Starzynski Bacchi

**Affiliations:** ^1^ Hospital das Clínicas da Faculdade de Medicina da Universidade de São Paulo (FMUSP), São Paulo, Brazil; ^2^ Hospital das Clínicas da Faculdade de Medicina da Universidade de São Paulo (FMUSP), Instituto de Psiquiatria, São Paulo, Brazil; ^3^ Laboratório de Neuroimagem em Psiquiatria (LIM21), Instituto de Psiquiatria, Hospital das Clínicas (HCFMUSP), Faculdade de Medicina, Universidade de São Paulo, São Paulo, Brazil

**Keywords:** zolpidem, substance use disorder, women, weekly follow-up, multidisciplinary approach, regulation

## Abstract

**Objective:**

This study presents a case series of five women with zolpidem dependence treated at the Drug Dependent Women Treatment Center (PROMUD), one of the first women-specific substance use disorder outpatient services in Latin America.

**Methods:**

This was an retrospective review of medical records of patients with a diagnosis of zolpidem dependence at the Institute of Psychiatry of Clinics Hospital of University of São Paulo between December 2021 and December 2023. Description of the cases followed the Case Report Statement, Checklist and Guidelines (CARE). The weekly zolpidem intake, comprising prescribed amounts and relapse episodes, was totaled to compute a mean daily dose. This was graphically illustrated to bring clinical insights.

**Results:**

The patients, aged 25-45 years, displayed escalating oral zolpidem doses (range: 60-900 mg/day), with adverse effects such as memory and social impairment, falls, seizures. Commonalities among cases included initiating zolpidem use for primary insomnia and withdrawal symptoms, including rebound insomnia, social impairment, and craving. History of physical and psychological abuse were reported. Comorbid psychiatric conditions, particularly eating disorders (n=3), recurrent depression (n=1), borderline personality disorder traits (n=1), and attention deficit hyperactivity disorder (n=1), were identified. Although zolpidem abuse often correlates with the concurrent abuse of other substances, none of the reported cases in this study exhibited other substance use disorders. Concurrent use of sedatives, especially benzodiazepines and levomepromazine, was observed (n=2).

**Conclusion:**

The surge in zolpidem prescriptions, driven by its perceived safety and low abuse potential compared to benzodiazepines, may lead to a global health issue of dependence. The medical community faces the challenge of managing this without standardized treatment protocols. Our case series underscores the effectiveness of the PROMUD program, which employs a multidisciplinary, women-specific approach with tailored group therapies and weekly psychiatric appointments to address and prevent relapse.

## 1 Introduction

In the 1980s, Z-drugs (i.e., zolpidem, zopiclone, zaleplon) were introduced to the market, under the necessity to improve the therapeutic arsenal for the management of insomnia (1). For the short-term treatment of this condition, benzodiazepine agents had been widely used for many decades, with significant adverse effects observed from this experience, primarily next-day sedation, daytime sleepiness, dependence, and withdrawal syndrome (2). The non-benzodiazepine hypnotics, on the other hand, were launched with the promise of providing safety in the short-term insomnia treatment (3).

Benzodiazepines and Z-drugs act on the gamma-aminobutyric acid receptor complex (GABA-A), resulting in significant inhibitory effects in the central nervous system (4). The GABA-A receptor complex has two main subtypes: ⍵1 (α1β1-3γ2 subunits), associated with sedative and amnesic effects, and ⍵2 (α2,3,5β1-3γ2 subunits), linked to antidepressant effects and anxiety control (3).

Zolpidem, a prototype of non-benzodiazepine hypnotics, binds with a high selectivity to the α1 subunit of the GABA-A receptor, in comparison to the α2, α3 and α5 subunits (5). Based on these pharmacokinetic aspects, zolpidem was expected to reduce the potential for abuse, propensity for tolerance, and withdrawal syndrome associated with the use of benzodiazepines, as a potent hypnotic with minimal anxiolytic effect.

The prescription of hypnotics non-benzodiazepines increased about 30-fold between 1993 and 2007 in the United States (6), outpacing both complaints of sleeplessness and diagnoses of insomnia. Globally (7), Z-drugs consumption continues to rise significantly every year, especially in higher-income countries. Regarding Brazil (8, 9), zolpidem is mentioned as one of the most sold and consumed psychotropic drugs during the COVID-19 pandemic, although there is a lack of evidence of the prevalence of zolpidem consumption at the population level and the increase in the prescription of this sedative in the Brazilian reality.

During the 1990s, zolpidem was believed not to have adverse effects related to tolerance and/or dependence (10–14), or to present a much lower risk of dependence compared to benzodiazepines (2, 14). Zolpidem intoxication is associated with adverse effects encompassing sleepwalking, sleep-related eating disorders, parasomnias, rebound insomnia, and even psychotic episodes. High doses can evoke psychostimulant responses, inducing sensations of well-being, euphoria, and loquaciousness. Prolonged exposure to high doses of zolpidem has been correlated with tolerance, social impairment, loss of control, intense cravings, and other withdrawal symptoms (15–18). Recently, the Brazilian government implemented stricter regulations regarding zolpidem, which could previously be dispensed without oversight for formulations containing up to 10 mg per unit. Going forward, a more controlled type of prescription will be required, available only to physicians registered with the local health surveillance agency, regardless of the dose prescribed (19).

Presently, we are witnessing an increase in report of cases and case series of zolpidem dependence (17, 20–25), complemented by a limited number of review articles (2, 17, 24).This crescenting evidence leads us to believe that the abuse and dependence on zolpidem are already emerging as a trend in recent years. However, there is a lack of quantitative evidence of zolpidem dependence, and most studies are still case reports. Moreover, until the present day, there is no published evidence of zolpidem dependence cases in Brazil and very few in Low-Middle income countries (26–29). The present study aims to describe a series of case reports of zolpidem dependence in an outpatient women-specific tertiary service for substance use disorder (SUD) in Brazil, the Drug Dependent Women Treatment Center (PROMUD).

## 2 Case presentation

### 2.1 Case 1

A 25-year-old female patient presented with a one year history of oral zolpidem abuse, which began after the distressing end of a 9-year relationship. History included bulimia in adolescence, previous use of clonazepam for anxiety control. The patient consumed alcohol in a non-abusive pattern and marijuana occasionally. Family history included SUD, specifically alcohol and cocaine, and a brother with severe depression and suicide attempts. She had a history of physical and psychological violence in her past relationship, as well as psychological abuse from her father (**Table 1**).

**Table 1:** Demographics, clinical and psychiatric characteristics, treatments, and outcomes of five patients with zolpidem dependence.

Clinical and Psychiatric Characteristics					
Age	25	28	36	32	45
Marital status	troubled relationships, married later	troubled relationships	married	single	married
Occupation	unemployed	healthcare worker	healthcare worker	unemployed	unemployed
Psychiatry comorbidities	Bulimia Nervosa, Anxiety	Anorexia nervosa	suspicion of borderline disorder, recurrent depression, ADHD	None	None
Psychiatric family history	Yes	Yes	Yes	Yes	No
Family substance use disorders	Yes	No	Yes	Yes	No
History of violence	Yes	No	Yes	No	Yes
Previous treatment attempts	1	1	1	0	2
Age at beginning	22	25	31	30	29
Time of zolpidem use	3 years	30 months	5 years	27 months	17 years
Time to develop dependence	2 years	6 months	2 years	20 months	7 years
Other substance use disorder	No	No	Yes (Nicotine)	No	No
Zolpidem dose					
Initial zolpidem daily dose	20 mg/d	600 mg/d	60 mg/d	40 mg/d	60 mg/d
Maximum consumption in one day	800 mg	1200 mg	60 mg	300 mg	400 mg
Maximum calculated mean daily dose*	400 mg/d	600 mg/d	60 mg/d	40 mg/d	185 mg/d
Intoxication Symptoms					
Excessive daytime sleepness	Yes	No	No	No	Yes
Dissociative nocturnal episodes	Yes	No	Yes	No	Yes
Falls	Yes	Yes	No	No	Yes
Memory loss	Yes	Yes	No	Yes	Yes
Attentional impairment	Yes	Yes	No	Yes	Yes
Withdrawal Symptoms					
Tolerance	Yes	Yes	Yes	Yes	Yes
Rebound insomnia	Yes	Yes	Yes	Yes	Yes
Social impairment	Yes	Yes	Yes	Yes	Yes
Lack of control over sedative consumption	Yes	Yes	Yes	Yes	Yes
Craving	Yes	Yes	Yes	Yes	Yes
Convulsive episodes	No	Yes	No	Yes	No
Previous medications					
Antidepressants	Fluoxetine 40 mg/d	Fluoxetine 60 mg/d	Sertraline 50 mg/d, Mirtazapine 15 mg/d, Venlafaxine 150 mg/d	–	Escitalopram 20 mg/d
Anticonvulsants	–	Valproic acid 1000 mg/d	–	–	–
Sedatives	Quetiapine 50 mg/d, Promethazine 50 mg/d, Levomepromazine 25 mg/d, Clonazepam 2 mg/d	Diazepam 240 mg/d	Diazepam 5 mg/d, Levomepromazine	Trazodone 150 mg/d	Trazodone 100 mg/d, Levomepromazine, Clonazepam 2-6 mg/d, Quetiapine 100 mg/d, Amitriptyline 125 mg/d
Others	Topiramate 100 mg/d	–	Naltrexone 50 mg/d, Ritalin 10 mg/d	–	–
Medications prescribed in PROMUD**					
Antidepressants	Fluoxetine 20 mg/d (1st-75th week)	Fluoxetine 60 mg/d (1st - 44th week)	Sertraline 50-150 mg/d (1st - 45th week)	Fluoxetine 20-40 mg/d (4th-25th week)	Escitalopram 20 mg/d (1st-10th week)
Anticonvulsants	–	Valproic acid 500-1000 mg/d (1st - 43th week)	–	–	–
Sedatives	Clonazepam 2 mg/d (3th - 27th week), Quetiapine 25-75 mg/d (14th - 63th and 74th - 75th week), Promethazine 25-50 mg/d (30 th - 63th week), Chlorpromazine 150 mg/d (64th - 65th week), Pericyazine 10-50 mg/d (Pericyazine 10-50 mg/d) (66th - 75th week)	Quetiapine 50-100 mg/d (1st - 15th and 23th -44th week), Chlorpromazine 25 mg/d (16th - 22nd week), Diazepam 10-60 mg/d (30th - 37th week)	Quetiapine 12,5-50 mg/d (1st -15th week), Levomepromazine 10-20 drops/d (6th - 25th week), Risperidone 1-2 mg/d (16th - 45th week)	Quetiapine 25-50 mg/d (1st-25th week)	Amitriptyline 100 mg/d (1st - 10th week), Diazepam 10 mg/d (1st - 4th week), Quetiapine 100-200 mg/d (1st - 10th week), Clonazepam 4 mg/d (5th-10th week)
Others	Topiramate 100 mg/d (63 weeks)	–	–	–	–
Multidisciplinary treatment					
Psychoeducation	Yes	Yes	Yes	Yes	Yes
Psychotherapy	Yes	Yes	Yes	Yes	Yes
Peer groups	Yes	Yes	Yes	Yes	Yes
Nutrition	Yes	Yes	Yes	Yes	Yes
Outcomes					
Time of follow-up	75 weeks	44 weeks	45 weeks	25 weeks	10 weeks
Number of relapses	21	12	0	3	2
Hospitalizations	1	2	0	0	1
Time of hospitalization	2 weeks	24 weeks, 4 weeks	–	–	8 weeks
Achieved abstinence?	Yes	Yes	Yes	No	No
Continued abstinent?	No	Yes	Yes	–	–

*The weekly zolpidem consumption, comprising prescribed amounts and relapse episodes, was totaled to compute a mean daily dose. **All prescribed medications were taken orally.

After the detachment from her ex-fiancé, she reported increasing her zolpidem consumption to 150 mg/day, distributed throughout the day. She reported falls, burns, sending inappropriate messages, episodes of forgetfulness, difficulty in memory retention, attention impairment, and excessive daytime sleepiness.

**Figure 1:**
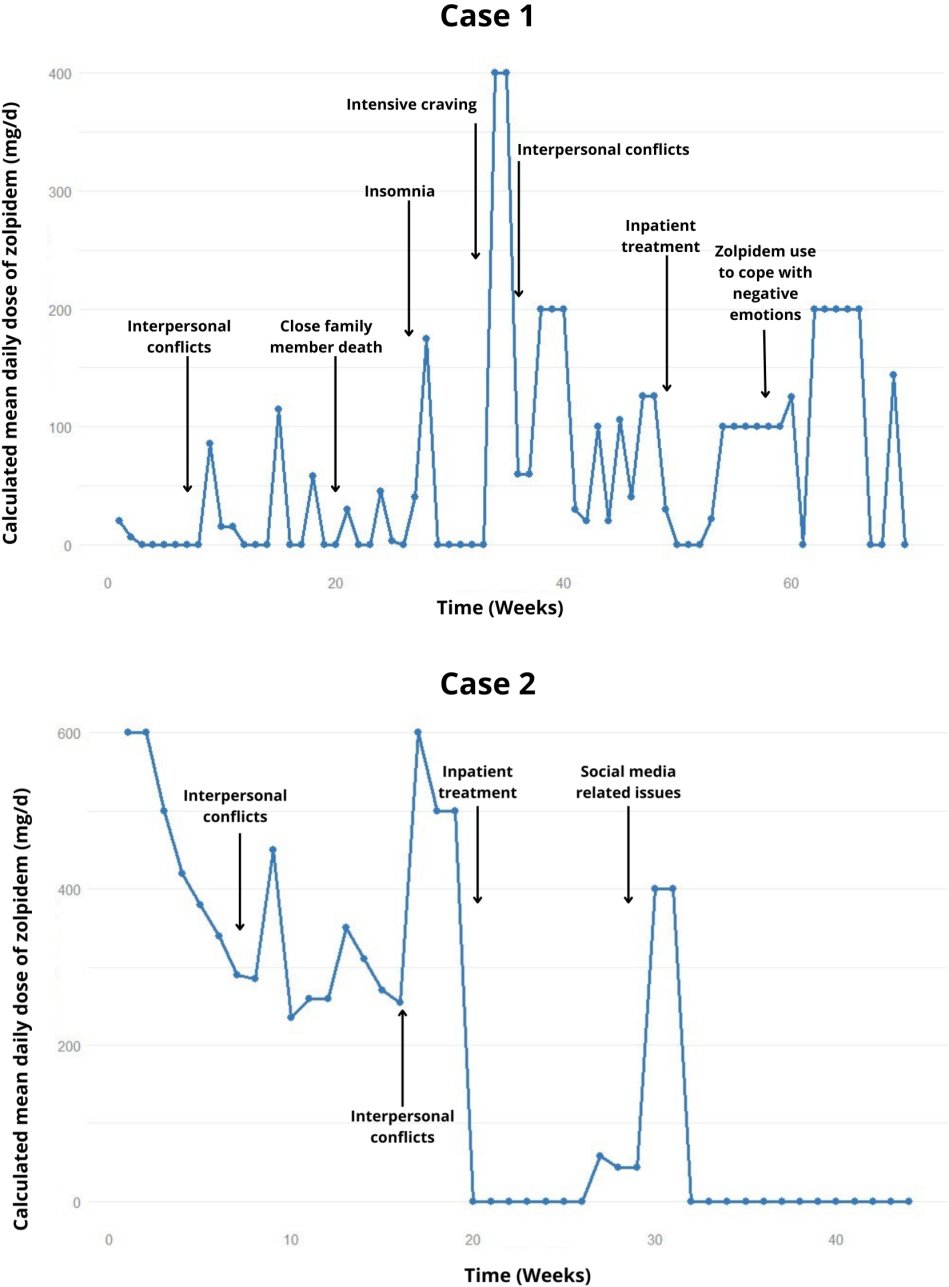
Weekly trend of the calculated mean daily dose of zolpidem, highlighting relapses and potential precipitating factors in the cases 1 and 2.

She was admitted using 20 mg/day of zolpidem, which was gradually discontinued and replaced by clonazepam (2 mg/day). The patient had 9 relapses until the tenth month of treatment, characterized by the ingestion of multiple tablets usually in a single day, after interpersonal conflicts. The use of zolpidem during these episodes ranged from 20-600 mg/day, distributed throughout the day (**Figure 1**).

After ten months, she was ingesting about 400-500 mg/day of zolpidem, with a peak intake of 800 mg in a single day. She also abused clonazepam, unable to specify the exact amount. Only after strict family surveillance, the patient managed to reduce zolpidem consumption to 20 mg/day, after the possibility of involuntary hospitalization was suggested.

In the subsequent months of treatment, she was ingesting between 20-40 mg/day of zolpidem and had 3 relapse episodes, ingesting about 400-600 mg, usually in a single day. Hospitalization was then indicated for control of the condition, lasting 1 month, successfully achieving abstinence. One week after hospitalization, she relapsed into zolpidem consumption and returned to the previous pattern of use, discontinuing her follow-up shortly thereafter.

### 2.2 Case 2

A 28-year-old female patient, healthcare worker, was admitted due to a two-year history of problematic oral zolpidem use. Following discharge from a private psychiatric facility, she began outpatient treatment. Psychiatric history included purging anorexia nervosa since adolescence, with two prior hospitalizations for this condition. She denied any other substance use (**Table 1**).

Initially, she complained of insomnia and was prescribed 10 mg/day of zolpidem, progressively increased dosage and noticed memory problems, three seizure episodes, a car accident while intoxicated, and a fall resulting in head trauma. She also had educational impairments with inattention and repeated absences. She was admitted consuming approximately 600-900 mg/day of zolpidem. Home hospitalization and a 15-day work leave were prescribed, with a maximum of 420 mg/day of zolpidem to avoid seizures.

In the following months, a strategy of reducing zolpidem intake by 10% per week was attempted. In the second month of treatment, she experienced withdrawal symptoms (tremor, sweating, cravings), but no seizures. When the patient was supposed to reach 210 mg/day, she confessed to actually consuming around 260 mg/day, self-prescribing additional amounts of zolpidem, which later led to a regression to 350 mg/day (**Figure 1**). Other relapses occurred, also attributed to interpersonal conflicts, and she returned to 500 mg/day. Thus, a weekly reduction of 100 mg/day of zolpidem dosage was attempted, reaching an approximate 300 mg/day. After these 5 months of outpatient treatment failure, and social exposure, a new hospitalization was opted for.

The second hospitalization lasted 1 month and the patient remained abstinent, but experienced relapses after one month of discharge. Initially with 200-300 mg on single days, escalating to 900-1200 mg/day relapse, following dissatisfaction related to social media. She was seen knocking down objects. Diazepam (60 mg/day) was introduced as a replacement for zolpidem. During this time, she was working night shifts and using the sedative up to 7 hours before work, showing little concern about working in this context and denying feeling intoxicated by the medication. The family opted for a new home hospitalization.

During the following 2 months, the patient remained abstinent from zolpidem, and there was progressive weaning of diazepam until complete cessation.

### 2.3 Case 3

A 36-year-old married female patient, healthcare worker, initiated outpatient follow-up during her pregnancy, presenting with a five-year history of oral zolpidem dependence. Psychiatric history includes suspicion of borderline disorder and recurrent depression, initially treated with venlafaxine and later with sertraline 50 mg/day, as well as attention deficit hyperactivity disorder, treated with Ritalin. Family history includes a half-brother with autism, a maternal uncle with schizophrenia, and multiple relatives with depression and alcoholism. She has a history of aggression towards her mother, roommates, and ex-boyfriend. She has used cocaine recreationally and occasionally consumes alcoholic beverages (**Table 1**).

She started taking the sedative 5 years prior, initially at 5 mg/day due initial insomnia, eventually reaching a consumption of 55 mg/day. Furthermore, she has experienced episodes of sleepwalking. In the first appointment, it was decided to reduce zolpidem to 40 mg/day, but it resulted in a worsening of depressive symptoms, leading to an increase in sertraline to 100 mg/day. Additionally, she was not attending prenatal appointments and returned using electronic cigarette (4000 puffs in 5-7 days).

**Figure 2:**
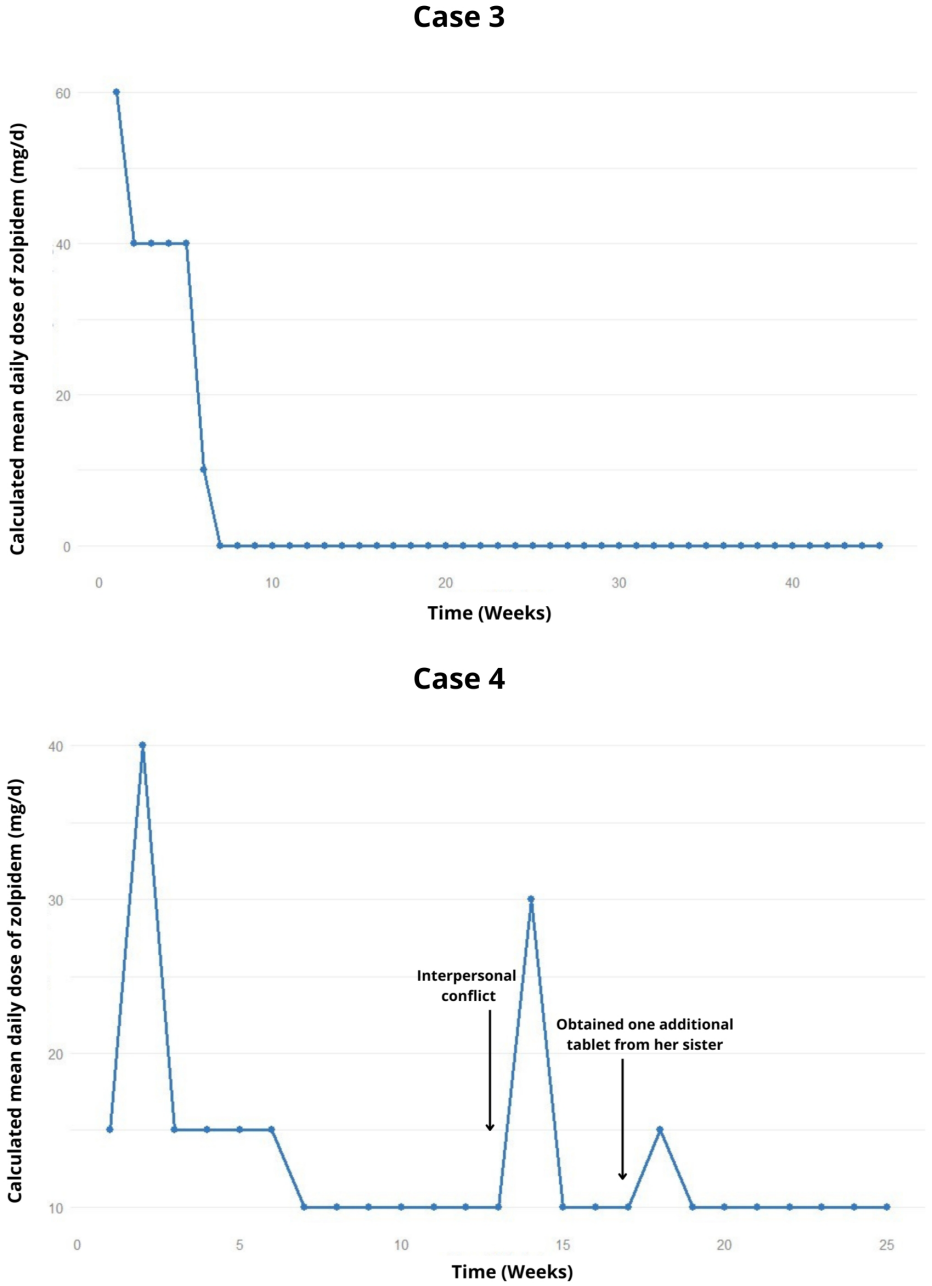
Weekly trend of the calculated mean daily dose of zolpidem, highlighting relapses and potential precipitating factors in the cases 3 and 4.

The patient then reduced zolpidem consumption to 10 mg/day (**Figure 2**), with the main complaints related to non-acceptance of pregnancy and thoughts about abortion or adoption. Therefore, the therapeutic plan included discontinuing zolpidem the following week, and gradual increase in sertraline dosage to 200 mg/day In the last recorded appointment to date, the patient remains abstinent from zolpidem for 9 months.

### 2.4 Case 4

A 32-year-old single female unemployed for a year, began outpatient treatment due to oral zolpidem abuse for two years. The patient occasionally uses alcohol, with no information about smoking or illicit drug use. Her history includes bariatric surgery resulting in significant weight reduction. Her family history reveals SUD among first-degree relatives (**Table 1**).

Zolpidem was initially offered by her sister (10 mg/day), to address insomnia following changes in the patient’s work schedule. She reported a growing need to increase intake to meet sleep demands, reaching a peak daily usage of 30 mg. She sought psychiatric treatment for zolpidem abuse, receiving a prescription for trazodone 150 mg/day and zolpidem 20 mg/day, with partial improvement. At admission she used 60 mg/day of zolpidem, and her mother later revealed she had taken over 300 mg/day in the past, leading to seizure episodes during abstinence.

The zolpidem dosage was gradually reduced; however two lapses occurred during interpersonal conflicts and when the patient took one additional tablet beyond the prescribed amount, obtained from her sister (**Figure 2**). After 6 months, the patient attempted to stop using zolpidem on her own. Therefore, it was decided to attempt a fixed reduction in zolpidem dosage to 5 mg/day. In the latest recorded appointment, the planned reduction had not been carried out, and the patient remains at the 10 mg/day dosage of zolpidem.

### 2.5 Case 5

A 45-year-old married female patient, healthcare worker, with a 10-year history of oral zolpidem dependence. Initially, she occasionally used clonazepam 2 mg/day following her mother’s death, later escalating to daily use. She frequently changed psychiatrists to obtain the medication, also exhibiting anxious symptoms. After a successful pregnancy, she discontinued clonazepam (6 mg/day). However, due to sleep-related complaints, she was prescribed zolpidem 10 mg/day and other sedatives without issues until 2013. She also reported physical violence by her husband (**Table 1**).

The patient self-increased the zolpidem dose, experiencing daytime drowsiness and rebound insomnia. Her second pregnancy, while using 90 mg/day, resulted in a stillborn at 36 weeks. Episodes of fainting and falls were also present, prompting her husband to recognize a SUD. An initial attempt to treat zolpidem dependence three years in the past was unsuccessful. The first hospitalization occurred one year prior to follow-up, tapering zolpidem with clonazepam (6 mg/day).

After a brief inpatient stay, the patient reported her husband’s refusal to purchase prescribed medications, resulting in intense cravings. Periods of up to 5 days without sleep and “disorganized behaviors” were reported, such as making inconsequential phone calls, and storing objects improperly. The patient exhibited low self-criticism regarding the resulting deficits. The initial outpatient prescription included zolpidem (10 mg/day); however, on some days of the week, she was using an additional 150-200 mg/day, with strict supervision from her husband.

**Figure 3:**
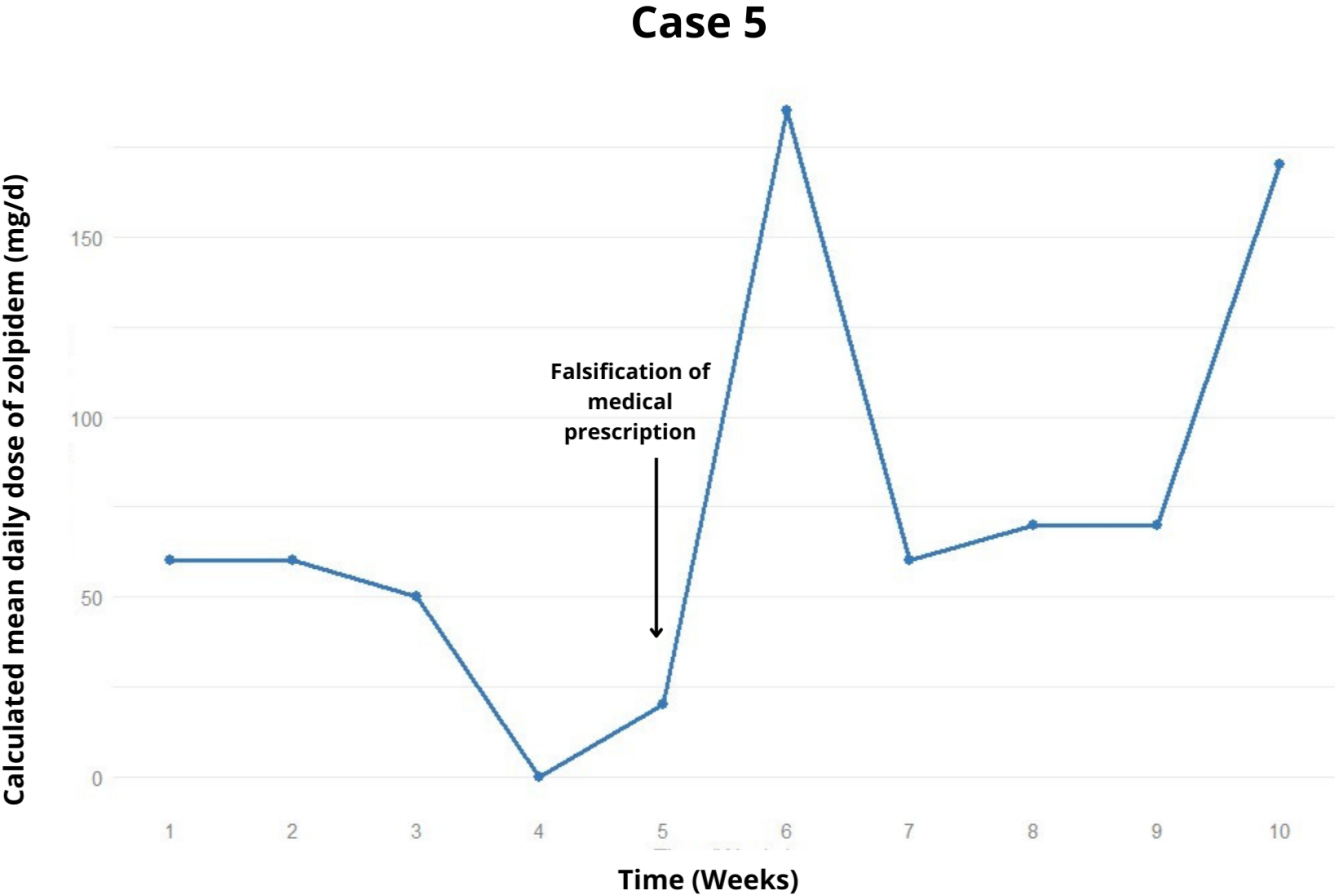
Weekly trend of the calculated mean daily dose of zolpidem, highlighting relapses and potential precipitating factors in the case 5.

After 6 weeks, a lapse occurred with approximately 300 mg of zolpidem for three days (**Figure 3**). Diazepam 10 mg/day was introduced, resulting in one week of zolpidem withdrawal. However, she continued exceeding the prescription, notably consuming up to 1200 mg. Consequently, clonazepam (4 mg/day) and quetiapine (200 mg/day) were introduced in an attempt to cease zolpidem consumption.

In the tenth week of treatment, despite being prescribed 20 mg/day of zolpidem, the patient was using 70-170 mg/day and still complained of intense insomnia, managing to sleep only 1 hour per night. She expressed a desire to increase both the zolpidem and clonazepam doses from her prescription. In the latest recorded appointment, the possibility of a second hospitalization was under discussion.

## 3 Discussion

In our case series, we described five cases of zolpidem dependence within a women-specific SUD outpatient treatment service at a tertiary hospital in Brazil (PROMUD). The dosages ranged from 60 mg/day to 900 mg/day prior to enrollment in the outpatient treatment program, with dependence developing within 6 months to 7 years. The age of beginning varied between 22 and 31 years old, notably young females. Three cases also involved misused of other sedatives, primarily clonazepam and levomepromazine.

Excessive daytime sleepiness (n=2), dissociative nocturnal episodes with and without binge eating (n=3), falls (n=3), memory loss, attentional impairment (n=4) were noted as effects of zolpidem intoxication. Regarding withdrawal symptoms, convulsive episodes were observed (n=2), while tolerance, rebound insomnia, social impairment, lack of control over sedative consumption and craving were evident in all patients. In three cases, previous traumatic experiences (physical and/or sexual and/or psychological abuse) were reported. Additionally, four cases had positive familial psychiatric backgrounds, including SUD (alcohol, cocaine, zolpidem), schizophrenia, autism, depression, and anxiety. Eating disorders were noted as a psychiatric background in three patients. Two patients were healthcare workers. Three cases required hospitalization at some point to continue the outpatient treatment.

While zolpidem abuse is frequently associated with concurrent abuse of other substances (i.e, alcohol, cocaine, heroin, cannabis) (18, 21, 30, 31, 32), none of the reported cases exhibited other SUD, with the exception of nicotine in one case. All cases initially began using zolpidem to address primary insomnia complaints, receiving official prescriptions without adequate long-term follow-up or consideration of first-line and safer medications for insomnia, ultimately leading to iatrogenic insomnia. Lugoboni et al. described at least two categories of zolpidem misusers. The first group comprises individuals who initiate zolpidem consumption for its hypnotic effects, subsequently developing tolerance to the sedative properties and progressively escalating zolpidem dosage until becoming abusers and dependent. The second category includes subjects, particularly those with a concomitant and/or previous SUD, mainly alcohol, who use zolpidem for recreational purposes.

All our patients reported initiating zolpidem consumption to achieve hypnotic effects. However, we also observed, in three documented cases, that these patients began to experience euphoria and a reduction in anxious symptoms upon chronic ingestion of zolpidem at high doses. Patients described feelings of “pleasure when taking the medication” or “a sense of well-being, even in the absence of sleep”, indicating a shift in the pattern of consumption: initially involving the use of the medication at high doses for its prescribed intention, but evolving towards the pursuit of recreational effects and a desire to “feel good” with zolpidem. These two distinct purposes characterize a phenomenon now known as “pharming” (33), defined as the misuse of both prescription medications, such as zolpidem, and over-the-counter drugs.

This transition may be attributed to the loss of selectivity of zolpidem to the α1 subunit of the GABA-A receptor at supratherapeutic dosages. In this context, zolpidem may interact with the α3 and α5 subunits, resulting in memory impairment; and with the α2 subunit, leading to anxiolytic effects, stimulation and euphoria (17, 24). In addition, interactions between the dopaminergic transmission and GABA have been purpoused (24, 31), suggesting that zolpidem could have a paradoxical amphetamine/cocaine-like effect at supratherapeutic doses.

Our findings underscore the risks associated with the indiscriminate prescription of zolpidem by clinicians, emphasizing the need for careful consideration of at-risk population subgroups for misuse/abuse and dependence. Specifically, this study observed the development of dependence in young women; however, other risk subgroups, such as alcohol-dependent and comorbidly-abusing patients (34), were not represented in our sample.

In the post-marketing phase of zolpidem, and in light of the increasing prevalence of Novel Psychoactive Substances misuse (35), real-world usage patterns appear to diverge significantly from those prescribed by physicians. Pharmacovigilance measures are now crucial, beginning with clinical observation and extending to research capable of establishing a solid level of evidence.

The European Medicines Agency, through its EudraVigilance database, has succeeded in quantifying adverse drug reactions associated with zolpidem over the past two decades. Notably, there has been a significant number of reports concerning misuse, abuse, dependence, withdrawal, as well as suicidal behavior and fatal outcomes (34). These data give further grounds to tighter regulations that are being implemented by the Brazilian government and other authorities around the world (36).

Our clinical experience has shown that sleep-inducing drugs and those that reduce impulsive behaviors, such as atypical antipsychotics, especially quetiapine, along with zolpidem replacement therapy with clonazepam, could be suitable therapeutic interventions, as previously described (37). Although inpatient treatment exercises a significant clinical and psychiatric compensatory function, particularly for individuals seeking euphoria or recreational use (17, 21, 24, 30,) we advocate for outpatient treatment as a more desirable approach to address dependence.

### 3.1 Strengths and limitations

To our knowledge, this is the first research focused on the longitudinal weekly follow-up of outpatients with zolpidem dependence, detailing relapses and the treatment strategy. Our limitations are the small number of patients and the short follow-up time.

## 4 Conclusion

We posit that the exponential rise in the prescription of zolpidem over the past two decades (16, 38, 39), coupled with the belief of a pharmacologically safer profile compared to benzodiazepines, with a minimal potential for abuse and/or dependency, have been pivotal factors to the exclusion of this health issue in a global scale. Thus, the entire medical community will face the challenge of treating zolpidem dependence, for which there is no standardized treatment protocol.

The reported patients exemplify the functioning of our women-specific SUD outpatient treatment (PROMUD): a program overseen by a multidisciplinary team, incorporating group therapies exclusively tailored to women and weekly appointments with a psychiatrist to ensure close monitoring. These appointments focus on identifying the factors sustaining consumption and crafting a relapse prevention strategy.

## Data Availability

The original contributions presented in the study are included in the article/supplementary material. Further inquiries can be directed to the corresponding author.
